# From Fishery Waste to Functional Adhesives: Milkfish (*Chanos chanos*) Skin Collagen–Polyvinylpyrrolidone Physically Crosslinked Biohybrid Adhesive for Sustainable Paper Bonding

**DOI:** 10.3390/polym18091121

**Published:** 2026-05-01

**Authors:** Kangsadan Boonprab, Jirawat Satiankomsorakrai

**Affiliations:** Department of Fishery Products, Faculty of Fisheries, Kasetsart University, Bangkok 10900, Thailand; jirawat.seng@gmail.com

**Keywords:** collagen, fish skin, adhesive, glue stick

## Abstract

Environmental concerns over plastic-based adhesives highlight the urgent need for biodegradable alternatives. This study transforms milkfish (*Chanos chanos*) skin waste from the fishery industry into a collagen–polyvinylpyrrolidone (PVP) biohybrid adhesive stick for paper bonding. Milkfish showed the highest adhesive strength among twenty species, requiring ≥213.7 mg/g hydroxyproline for optimal performance. Type I collagen was confirmed via Fourier transform infrared (FTIR) and amino acid composition, and the extraction yield reached 68.82%. The fish skin collagen–PVP glue stick demonstrated paper adhesion and physicochemical properties comparable to starch-based and commercial glues, with lower hardness and more dry adhesive per unit area. Sensory evaluation using quantitative descriptive analysis revealed no significant differences (*p* < 0.05) compared to commercial glue sticks, except for increased glue consumption and reduced shape retention. The shelf life exceeded 70 days. Collagen adhesive from fish skin offers comparable efficiency to chemical and other bio-based adhesives, providing a sustainable solution that promotes the circular economy and green innovation.

## 1. Introduction

The global fishery industry generates substantial amounts of by-products—such as fish heads, skins, and bones—after primary processing. Without proper management, these residues contribute to environmental pollution and increase disposal costs. Their valorization into high-value, natural-based products offers a sustainable approach to waste reduction, climate change mitigation, and resource efficiency, while creating added economic value [1,2,3].

Accordingly, the green market is expanding rapidly, with a projected CAGR (Compound Annual Growth Rate) of over 19.5% between 2023 and 2032 [4], driven by growing demand for sustainable solutions. A key emerging trend is the circular economy, which emphasizes reuse, recycling, and remanufacturing. This concept is central to the bio-based industry, where renewable materials are harnessed to reduce waste and environmental impact across sectors such as packaging, textiles, and bioplastics. This work highlights the utilization of fish skin as a sustainable biomaterial within the framework of the circular economy, capitalizing on its predominant collagen content, which exhibits intrinsic adhesive properties. Previous research on 20 freshwater and marine species identified *Chanos chanos* skin as the most promising source for bioadhesive production [5]. The collagen structure of *C. chanos* has also been previously characterized using SDS–PAGE [5]. Here, a collagen-based glue stick derived from milkfish (*C. chanos*) skin for paper adhesion applications was developed. Driven by growth in the green market and a projected expansion of the global glue stick market to USD 2.3 billion by 2033 (CAGR 5.5% from 2026 to 2033) [6], there is a rising demand for non-toxic, biodegradable adhesives. This work aligns with circular economy principles by valorizing fishery by-products and advancing sustainable adhesive materials.

Adhesives are essential in daily life, with glue sticks widely used in schools, offices, and the packaging industry due to their convenience and ease of application. However, the vast majority of commercially available glue sticks as well as various patents are derived from petroleum-based synthetic polymers, such as polyvinylpyrrolidone, polyurethane, and polyvinylactate, which are non-biodegradable and contribute significantly to environmental pollution when discarded improperly [7,8,9]. In recent years, the increasing awareness of environmental sustainability and circular economy principles has driven research towards the development of eco-friendly, bio-based adhesives. Among bio-based adhesives, natural protein-based glues such as whey protein, seaweed extract, and starch adhesives have received growing attention due to their biodegradability and renewability [10,11,12]. However, these natural glues often exhibit limitations in terms of adhesion strength, water resistance, and storage stability [13]. For example, starch-based glue tends to have lower bonding performance compared to its synthetic counterparts and is prone to microbial spoilage [14]. As a result, the demand for innovative formulations that enhance the functionality and practicality of natural adhesives remains high.

To address the limitations of pure natural adhesives, blending with synthetic polymers to create biohybrid systems has emerged as an effective strategy. Recent studies have explored biohybrid adhesives based on collagen–polymer systems, where collagen is combined with polymers such as polyvinyl alcohol (PVA) and chitosan to enhance mechanical strength, stability, and biocompatibility through intermolecular network formation [15,16,17]. These systems have shown promising properties in biomedical and hydrogel applications, including improved flexibility and adhesion performance. However, collagen-based materials still suffer from limitations such as poor intrinsic mechanical stability and high moisture sensitivity, and most studies have focused on scaffolds or hydrogels rather than functional adhesive applications [15]. In addition, the use of collagen derived from sustainable sources, such as fish skin by-products, remains limited. Therefore, the relationship between material composition and adhesive performance in such biohybrid systems remains unclear, highlighting an important research gap.

Polyvinylpyrrolidone (PVP) is a synthetic, water-soluble polymer known for its excellent film-forming properties, biocompatibility, and ability to enhance the adhesion performance and stability of biopolymers [9]. It is known to be used as an adhesive ingredient for glue sticks, as evidenced by its presence in various patents [18,19,20]. In addition, from a theoretical perspective, PVP contains a lactam group capable of forming intermolecular hydrogen bonds with functional groups in collagen (e.g., –NH and –OH), promoting molecular compatibility and network formation. These interactions enhance cohesion, film integrity, and overall adhesion performance of the polymer system [21,22]. Therefore, the incorporation of PVP is expected to improve the structural stability and adhesive properties of collagen-based materials, which are critical factors governing the mechanical stability and performance of collagen-based systems [23]. However, studies integrating fish collagen and PVP into a solid glue stick format suitable for paper bonding have not been reported to date.

Therefore, this study aims to valorize milkfish (*C. chanos*) skin, an underutilized marine by-product, as a sustainable source of collagen glue and to develop a novel biohybrid collagen–PVP adhesive stick for paper bonding applications. The objectives are (1) to characterize the physicochemical properties of the extracted collagen glue, including its functional groups (FTIR analysis) and amino acid composition; (2) evaluate the effect of pH on the adhesive properties of milkfish glue; (3) formulate a biohybrid glue stick by blending collagen with PVP; and (4) evaluate its adhesion performance, shelf life, and comparative properties against conventional starch-based and commercial synthetic glue sticks. The findings are expected to contribute to the development of practical, eco-friendly adhesives that promote waste valorization and align with global sustainability goals.

## 2. Materials and Methods

### 2.1. Materials

Fresh fish, including milkfish (*Chanos chanos*) and other representative freshwater and marine species (*Chanos chanos*, *Oreochromis niloticus*, *Psettodes erumei*, *Micronema bleekeri*, *Channa striata*, *Epiephelus malabaricus*, *Priacanthus tayenus*, *Lethrinus lentjan*, *Oreochromis* sp., *Barbonymus gonionotus*, *Lutjanus lineolatus*, *Nemipterus hexodon*, *Sphyraena jello*, *Xenentodon cancila*, *Liza vaigiensis*, *Anabas testudineus*, *Thunnus tonggol*, *Rastrelliger brachysoma*, *Pomadasys hasta*, and *Scomber japonicus*), were obtained from local markets and aquaculture farms in Bangkok and Prachuap Khiri Khan Province, Thailand. Samples were transported to the laboratory in insulated polystyrene containers with ice (4–10 °C). Upon arrival, fish skins were manually separated, rinsed with distilled water, and stored at −20 °C until further analysis. Polyvinylpyrrolidone (PVP K85–95), glycerol, glyceryl monostearate, stearic acid, sodium hydroxide, hydroxyproline, and collagen were purchased from Sigma-Aldrich (St. Louis, MO, USA), Carol Erba Reagent (Emmendingen, Germany), or KemAus (Cherrybrook, NSW, Australia). All other reagents used were of analytical grade. Commercial glue sticks (OfficeMate Co., Ltd., Bangkok, Thailand) and starch-based glue sticks (cassava starch adhesive) were used as comparative samples.

### 2.2. Extraction of Collagen-Based Adhesive from Milkfish and Other Species Ski

**Figure 1:**
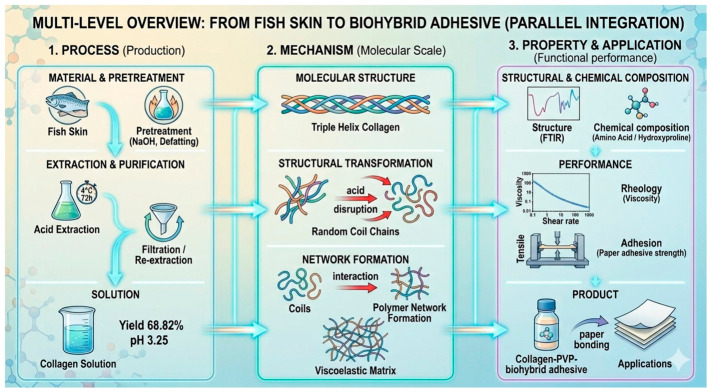
Schematic of process–structure–property relationships in collagen-based adhesive derived from fish skin, highlighting its application as a collagen–PVP biohybrid adhesive for paper bonding.

Fish skins were manually cleaned, defleshed, and cut into small pieces (1 cm × 1 cm). Non-collagenous proteins and pigments were removed by soaking the skins in 0.1 N NaOH for 6 h, with solution replacement every 3 h. Samples were rinsed twice with distilled water and subsequently defatted in 10% butyl alcohol for 24 h, followed by two washes with distilled water. Collagen extraction was conducted by immersing the pretreated skins in 0.5 M acetic acid (1:2 *w*/*v*) at 4 °C for 72 h. The resulting viscous mixture was filtered through four layers of muslin cloth to separate soluble collagen. The residual skin was re-extracted under identical conditions, and the filtrates were combined to obtain the crude collagen adhesive [5]. The process flow is illustrated in Figure 1.

### 2.3. Preparation of Collagen–PVP Adhesive Stick (Formulation FAS)

A collagen–PVP glue stick formulation was developed using the crude collagen extract. The optimized formulation (FAS: 10% PVP, 0% NaCl) comprised 5 g of collagen extract mixed with 25 mL of distilled water, 2.5 g of PVP K85–95, 5 g of glycerol (as plasticizer), 1.33 g of glyceryl monostearate (as emulsifier), 2 g of stearic acid (as consistency modifier), and 3.7% *w*/*w* NaOH (as neutralizing and stabilizing agent). Each ingredient was added sequentially under magnetic stirring at 100 rpm until homogeneous. The mixture was refluxed at 65–70 °C for 1 h, yielding a white viscous solution, and then poured into molds and cooled at room temperature (30–37 °C) for 12 h to form solid adhesive sticks. The collagen extract used in the formulation was obtained exclusively from milkfish (*C. chanos*) skin. The adhesive was prepared using a fixed optimized formulation, and no experimental variables were varied.

### 2.4. Characterization of Fish Skin Adhesive, Collagen, and Adhesive Stick

#### 2.4.1. Fourier Transform Infrared Spectroscopy

Fourier transform infrared (FTIR) spectra were recorded using an FTIR spectrometer (Agilent Cary 630/Agilent 5500 FTIR, Agilent Technologies, Santa Clara, CA, USA) equipped with an attenuated total reflectance (ATR) accessory. Samples were scanned over the wavenumber range of 4000–400 cm^−1^ at a resolution of 4 cm^−1^, with 32 scans averaged per spectrum. Background spectra were collected prior to each measurement. All spectra were baseline-corrected and normalized before analysis. Spectral peaks corresponding to amide I, II, and III bands were analyzed to confirm collagen integrity and interactions with PVP.

#### 2.4.2. Color Measurement

Color measurements (L*, a*, b*) were conducted using a spectrophotometer (UltraScan VIS, HunterLab, Reston, VA, USA) with a small area view (SAV) aperture under illuminant D65 and a 10° observer. Measurements were performed in triplicate, and results were expressed as mean ± SD.

#### 2.4.3. Moisture Content, pH, and Melting Point

Moisture content was determined gravimetrically at 105 °C to constant weight [24]. pH was measured by dissolving 1 g of the sample in 10 mL of distilled water using a pH meter (Mettler Toledo Seven Compact). The melting point was determined using the open-ended capillary tube method by heating the sample at 1–2 °C min^−1^ and recording the temperature range at which the material softened and completely melted [25]. This method is commonly used to assess the thermal transition behavior of polymeric and bio-based materials.

#### 2.4.4. Hardness

Texture analysis was performed using a texture analyzer (TA-HD plus, Stable Micro Systems, Godalming, UK) in compression mode equipped with a cylindrical probe (SMS P/2, 2 mm diameter). Glue stick samples were exposed by advancing the stick 3 mm above the container. Measurements were conducted at a test speed of 20 mm min^−1^ with a penetration depth of 3 mm, and hardness was recorded as the maximum force (N) during compression.

#### 2.4.5. Proximate Analysis

Proximate composition (moisture, ash, fat, protein, and carbohydrate) was determined according to standard AOAC methods [24]. Moisture content was measured via oven drying using a hot air oven (Memmert UNB400, Memmert GmbH + Co. KG, Schwabach, Germany). Ash content was determined via incineration in a muffle furnace (Vulcan A130, Yucaipa, CA, USA). Fat content was analyzed by means of Soxhlet extraction (Soxtec systemHT 1043 Extraction Unit, FOSS Analytical AB, Höganäs, Sweden). Protein content was determined using the Kjeldahl method with a digestion and distillation unit (Buchi Distillation Unit Type B-323, BÜCHI Labortechnik AG, Flawil, Switzerland), applying a nitrogen conversion factor of 6.25. Carbohydrate content was calculated by difference.

### 2.5. Adhesion Performance Evaluation

#### 2.5.1. Initial Adhesion Time

Initial adhesion was evaluated as early-stage peel strength measured according to ASTM D2979-16 (Withdrawn 2019) [26] at short setting times (20–140 s), reflecting the initial bonding performance of the adhesive on paper substrates. Although ASTM D2979-16 has been withdrawn, it remains a reference method for probe tack evaluation due to the absence of a direct replacement standard. Initial adhesion was expressed as the average force (N).

#### 2.5.2. Peeling Strength

Peel strength was measured following ASTM D2979-16 (Withdrawn 2019) [26]. Although ASTM D2979-16 has been withdrawn, it remains a reference method for probe tack evaluation due to the absence of a direct replacement standard. The adhesive was uniformly applied between paper substrates and conditioned under controlled bonding conditions. A 180° peel test was conducted using a texture analyzer (Stable Micro Systems TA-HD) equipped with HD tensile grips. The crosshead speed was maintained at 152.4 mm min^−1^. Peel strength was expressed as the average force required to separate the bonded substrates (N).

#### 2.5.3. Wet and Dry Adhesive per Unit Area

Wet and dry adhesive contents were determined according to ASTM D3121-17 [27] with minor modifications. Adhesive was applied onto a known paper area and weighed to obtain wet mass. The samples were then dried at 100 ± 5 °C for 14 h to constant weight for dry mass determination. The results were expressed as adhesive mass per unit area (g cm^−2^). All analyses were performed in triplicate.

### 2.6. Amino Acid Composition Analysis of Collagen-Based Adhesive from Milkfish Skin Using High Performance Liquid Chromatography (HPLC)

Amino acid composition of the adhesive was analyzed using HPLC. Samples, a collagen-based adhesive from milkfish skin, were hydrolyzed with 6 M HCl at 110 °C for 24 h and neutralized with 1.5 M NaOH. The hydrolysates were derivatized using borate buffer (pH 9.0) and AccQ-Fluor reagent, followed by heating at 55 °C for 10 min. A 10–20 µL aliquot was injected into an HPLC system (Waters 600E gradient pump with 717 Plus autosampler, Waters Corporation, Milford, MA, USA) equipped with a fluorescence detector (Jasco FP-920, JASCO Corporation, Tokyo, Japan). Separation was performed using the AccQ-Tag chemistry package with a gradient mobile phase system (Waters, Milford, USA). Detection was carried out at excitation/emission wavelengths of 250/395 nm. The procedure followed [28].

### 2.7. Determination of Hydroxyproline Content in Collagen-Based Adhesive from the Skins of Milkfish and Other Fish Species

Hydroxyproline standard solutions (0–60 ppm) were prepared from a 1000 ppm stock solution stored at 4 °C. Collagen samples (1 g) were hydrolyzed with 6 M HCl at 121 °C for 15 min, followed by decolorization with activated carbon, filtration, and neutralization to pH 6.0–6.5. The final volume was adjusted to 10 mL. Hydroxyproline content was determined based on a modified method of [29]. A calibration curve was constructed using standard solutions, and linear regression analysis showed good linearity with R^2^ ≥ 0.9990. For analysis, 0.1 mL of the sample solution was mixed with 0.2 mL of isopropanol and 0.1 mL of oxidant solution (7% *w*/*v* chloramine T in acetate/citrate buffer, pH 6.0, at a 1:4 *v*/*v* ratio). The mixture was vortexed and then reacted with 1.3 mL of Ehrlich’s reagent. The reaction mixture was incubated at 60 ± 0.5 °C for 25 min, cooled in ice water for 2–3 min, and diluted with 5 mL of isopropanol. The absorbance was measured at 558 nm using a UV-Visible spectrophotometer (Shimadzu UV-1700, Shimadzu Corporation, Kyoto, Japan).

### 2.8. Quantitative Descriptive Analysis (QDA) for Consumer Acceptance

A trained panel of 10 assessors evaluated sensory and functional attributes of the glue sticks using a fifteen-point intensity scale (1 = very weak; 15 = very strong). The following parameters were evaluated:

- **Pre-use appearance:** visual turbidity, surface smoothness, structural homogeneity.
- **In-use performance:** application smoothness, application hardness, glue consumption per application, coating uniformity.
- **Post-use bonding performance:** repositionability, bonding strength, and shape retention.

Commercial adhesive sticks were included as controls. All samples were coded with random three-digit numbers and presented in a randomized order under controlled lighting conditions (ISO 11035:1994) [30].

### 2.9. Statistical Analysis

All analyses were conducted in triplicate (*n* = 3), except for amino acid analysis for collagen-based adhesive from milkfish skin using HPLC, which was performed in duplicate (*n* = 2), and QDA, which involved 10 panelists. The results are presented as mean ± standard deviation (SD). Statistical differences among treatment groups were evaluated using one-way analysis of variance (ANOVA) conducted in Microsoft® Excel® 2019 (Version 2205, Build 16.0.15225.20278, 64-bit) (Product ID: 00405-32438-96064-AAOEM). When significant differences were detected, Duncan’s multiple range test was applied to compare group means, with statistical significance defined at *p* < 0.05.

### 2.10. Use of AI Tools for Graphical Preparation

During the preparation of this manuscript, the authors used Gemini to generate the graphical abstract and Figure 1, as well as parts of Figure 5. The outputs were reviewed, edited, and verified by the authors, who take full responsibility for the content.

## 3. Results and Discussion

**Characteristic of milkfish skin.** Chemical composition of milkfish skin: Proximate analysis was conducted to evaluate the potential of milkfish (*C. chanos*) skin as a protein-rich source for collagen extraction for adhesive applications. The skin comprised moisture (73.70 ± 0.01%), ash (0.210 ± 0.07%), fat (1.614 ± 1.34%), protein (10.65 ± 0.38%), and carbohydrates (12.87 ± 1.58%). The relatively high protein and carbohydrate contents suggest that milkfish skin is a promising raw material for bioadhesive production, potentially contributing to the mechanisms underlying adhesive functionality [31,32]. Carbohydrate content in fish tissues is generally low but variable, with values of ~4–5% reported in milkfish skin [33]. In this study, carbohydrate was estimated by difference, a method prone to overestimation due to cumulative analytical uncertainties [24]. In addition, contributions from collagen-associated glycoproteins and extracellular matrix components may account for part of this fraction; therefore, the values should be interpreted with caution [34]. Adhesion in biohybrid systems is primarily governed by intermolecular interactions (e.g., hydrogen bonding and van der Waals forces) and cohesive network formation within polymer matrices [35], whereas carbohydrate-related components do not directly contribute to adhesion but may play a secondary role in modulating hydration and interfacial conditions [36].

**Characteristics of the milkfish skin adhesive.** The milkfish (*C. chanos*) skin adhesive demonstrated physicochemical and adhesive properties comparable to traditional animal-derived adhesives, underscoring its potential as a sustainable bio-based alternative (Table 1). Its transparent gel-like appearance and viscosity were similar to animal hide adhesive but higher than bone adhesive, reflecting favorable rheological characteristics for glue applications [37,38]. The paper adhesion strength (19.3 × 10^−2^ MPa at 60 min) was within the performance range of conventional glues, confirming its suitability for paper bonding [39]. The adhesive exhibited higher moisture content than both hide and bone adhesives, which may account for its gel consistency, whereas lower ash, fat, and pH values indicated a cleaner composition with fewer impurities. Protein and carbohydrate levels were comparable across adhesives, supporting the role of collagen and polysaccharides as primary contributors to adhesion [40,41]. Moreover, the milkfish glue showed a residue after evaporation of 2.76% and a high extraction yield of 68.82%, demonstrating efficient collagen recovery. Collectively, these findings reinforce fish processing by-products as a promising raw material for bioadhesive development, advancing waste valorization within a circular economy framework [42].

**Figure 2:**
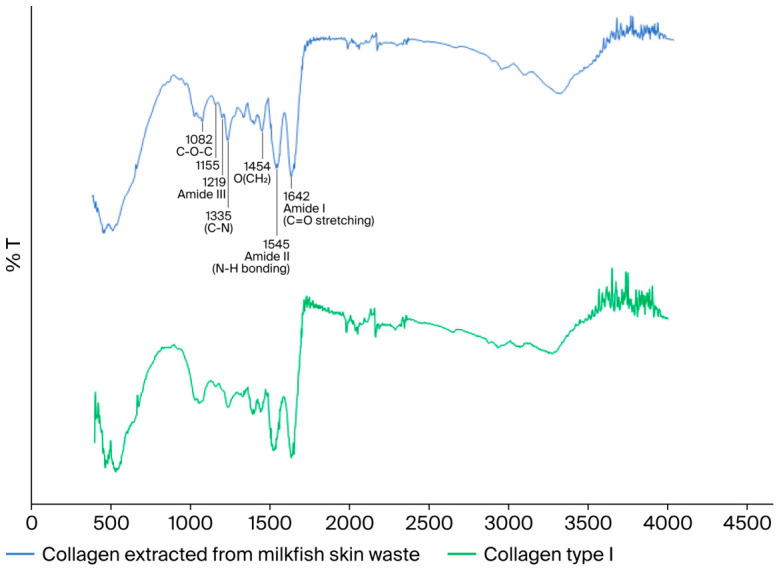
FTIR spectrum of collagen extracted from milkfish skin waste. This figure was created in Microsoft® Excel® 2019 (Version 2205, Build 16.0.15225.20278, 64-bit) (Product ID: 00405-32438-96064-AAOEM) and Microsoft® PowerPoint® 2019 MSO (Version 2508 Build 16.0.19127.20302) 64-bit (Product ID: 00405-32438-96064-AAOEM).

**FTIR spectroscopy of the collagen structure of the milkfish skin adhesive.** The FTIR spectrum profile of the collagen extracted from milkfish skin is shown in Figure 2. The experimental results revealed absorption peaks at 1642, 1545, 1454, 1335, 1238, 1219, 1155, and 1082 cm^−1^. These peaks correspond to the characteristic FTIR absorption bands of Type I collagen previously reported by Belbachir et al. (2009) [44], which appeared at 1659, 1555, 1454, 1403, 1340, 1282, 1240, 1203, 1160, 1079, and 1035 cm^−1^. The absorption bands at 1035 and 1079 cm^−1^ are attributed to the stretching vibrations of (C–O) and (C–O–C), indicating the presence of carbohydrate moieties. Meanwhile, the peaks at 1454, 1403, 1340, 1282, 1240, and 1203 cm^−1^ may correspond to the O(CH_2_), O(CH_3_), C–N, and O(N–H) bonds in the collagen structure. The amide I and amide II bands, observed at 1659 and 1555 cm^−1^, respectively, are related to the degree of molecular order in the triple-helix structure of collagen, arising from the stretching vibration of C=O and the bending vibration of N–H [45]. The FTIR analysis provided strong evidence for the successful incorporation of fish skin-derived collagen into the adhesive formulation. The observed peaks at 1642 and 1545 cm^−1^ correspond to amide I (C=O stretching) and amide II (N–H bending) vibrations, respectively, which are characteristic signatures of the triple-helical structure of Type I collagen [44,45]. The presence of these bands indicates that the native secondary structure of collagen was largely preserved after extraction and blending with polyvinylpyrrolidone (PVP), suggesting that the formulation process did not induce significant denaturation. In addition, the absorption peaks at 1035–1079 cm^−1^, attributed to C–O and C–O–C stretching, revealed the coexistence of carbohydrate moieties and PVP backbone interactions. These findings imply hydrogen bonding and molecular entanglement between collagen and PVP chains, which likely enhance adhesive cohesion and water retention capacity. Similar molecular interactions between protein-based polymers and synthetic hydrophilic matrices have been reported to improve the viscoelastic properties and film-forming behavior of bioadhesives [46,47,48,49]. The preservation of amide I and II peaks, along with the shift in carbohydrate-associated regions, indicates that PVP acts not only as a stabilizer but also as a computerizing agent, improving the interfacial bonding and mechanical integrity of the collagen-based adhesive. This synergistic molecular interaction provides a scientific rationale for the superior flexibility and application smoothness observed in the glue stick formulation compared to conventional commercial adhesives [49]. FTIR analysis indicates the presence of collagen and the potential for collagen–PVP interactions in the formulation, supporting its viability for further development into an adhesive stick.

**Figure 3:**
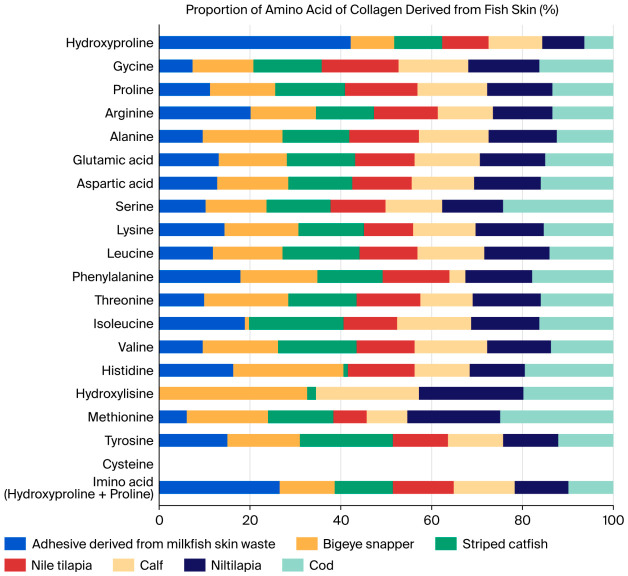
Proportion of amino acid content of collagen derived from fish skin (%) in a variety of fish species. Collagen-based adhesive from milkfish skin (this study) was compared with literature values from bigeye snapper [50], striped catfish [51], Nile tilapia [52,54], calf skin [53], and cod skin [54]. This figure was created in Microsoft® Excel® 2019 (Version 2205, Build 16.0.15225.20278, 64-bit) (Product ID: 00405-32438-96064-AAOEM).

**Amino acid composition of milkfish skin adhesive.** To further elucidate the structural characteristics relevant to adhesive function, the amino acid composition of the milkfish collagen adhesive was subsequently analyzed. The amino acid composition of the milkfish (*C. chanos*) skin-derived adhesive is presented in Figure 3. The adhesive was found to contain the following amino acids: hydroxyproline, glycine, proline, arginine, alanine, glutamic acid, aspartic acid, serine, lysine, leucine, phenylalanine, threonine, isoleucine, valine, histidine, methionine, tyrosine, and cysteine, with mean contents (mg per 100 g dry weight of glue; *n* = 2) of 554.1, 260.0, 144.0, 137.0, 123.0, 113.0, 69.0, 44.0, 44.0, 30.0, 26.0, 26.0, 21.0, 21.0, 11.7, 6.0, and 0.0, respectively. The amino acid profile showed a predominance of hydroxyproline, proline, glycine, alanine, and glutamic acid, which are typical of collagen-based proteins. The compositional pattern closely resembled that of collagens derived from other fish species, such as bigeye snapper [50], catfish [51], tilapia [52], calf skin [53], and gelatins from Nile perch and cod skin [54]. Consistent with previous findings [50,55], methionine, isoleucine, and tyrosine were present in low amounts, while cysteine was undetectable, as Type I collagen generally contains less than 0.2% cysteine [51]. A distinctive feature of the milkfish-derived adhesive was its high hydroxyproline content, whereas most other reported fish collagens exhibit glycine as the most abundant amino acid. This elevated hydroxyproline level suggests enhanced stability of the triple-helix structure, which may contribute to the adhesive’s bonding performance. The amino acid composition of the milkfish (*C. chanos*) skin-derived adhesive reflects the structural characteristics of Type I collagen, in which glycine, proline, and hydroxyproline dominate the sequence, forming a repeating (Gly–X–Y) motif responsible for triple-helix stability. The notably high hydroxyproline content observed in this study suggests a strong contribution to intramolecular hydrogen bonding, enhancing both the rigidity and cohesive strength of the collagen network. Such stabilization is critical for maintaining film-forming ability and adhesion performance under ambient conditions. Compared to collagens from other fish species, the elevated hydroxyproline proportion may explain the superior adhesive strength observed in milkfish glue. Previous studies have demonstrated a direct correlation between hydroxyproline levels and the tensile and thermal stability of collagen-based materials [56,57]. Therefore, the amino acid composition not only confirms the structural integrity of the extracted collagen but also provides a biochemical basis for its promising performance as a bio-based adhesive precursor. This evidence supports the further engineering of collagen–PVP hybrid glue sticks with optimized physicochemical and bonding properties.

**Hydroxyproline content and adhesive performance of milkfish skin adhesive.** As shown in Table 2, the hydroxyproline content [mean ± SD, mg/g dry weight of glue; *n* = 3] did not correlate linearly with paper bonding strength measured at 60 min, reflecting the complex nature of adhesive behavior. Among the samples, glue derived from *Epinephelus areolatus* exhibited the highest hydroxyproline content (705.6 ± 38.1 mg/g), followed by *Barbonymus gonionotus* (698.4 ± 69.7 mg/g) and *Psettodes erumei* (559.0 ± 85.8 mg/g). Notably, fish skins that yielded adhesives (ranked 1–12) had hydroxyproline levels ranging from 213.7 ± 58.5 to 705.6 ± 38.1 mg/g, whereas skins that did not produce adhesive properties (ranked 13–20) contained less than 213.7 ± 58.5 mg/g. These findings indicate that hydroxyproline content is a critical determinant for adhesive functionality, with a threshold of ≥213.7 ± 58.5 mg/g required to achieve glue-like behavior. Interestingly, hydroxyproline levels and adhesive performance were independent of fish habitat (freshwater versus marine). The study examined sixteen species, including freshwater fishes such as *Oreochromis niloticus*, *Micronema bleekeri*, *Channa striata*, *Barbonymus gonionotus*, *Xenentodon cancila*, and *Anabas testudineus*, as well as marine species such as *C. chanos*, *Psettodes erumei*, *Epinephelus areolatus*, *Selar crumenophthalmus*, *Lethrims lentjan*, *Lujanus lineolatus*, *Nemipterus hoxodon*, *Sphyraena jello*, *Liza vaigiensis*, *Thunnus tonggol*, *Rastrelliger brachysoma*, *Pomadasys hasta*, and *Scomber japonicus*. To our knowledge, this comprehensive quantification of hydroxyproline across sixteen fish species has not been previously reported; prior studies were limited to *Oreochromis niloticus*, *Psettodes erumei*, and *Nemipterus* spp. [55]. The data indicate that hydroxyproline content is a key biochemical determinant of adhesive functionality in fish skin-derived glues. Skins yielding adhesives consistently contained ≥213.7 ± 58.5 mg/g hydroxyproline, whereas non-adhesive samples were below this threshold. Although hydroxyproline levels did not correlate linearly with bonding strength, elevated hydroxyproline likely enhances triple-helix stability, promoting cohesive network formation and effective paper adhesion [58,59,60]. Notably, this pattern was independent of fish habitat, encompassing both freshwater and marine species, highlighting the universality of hydroxyproline as a predictive marker for adhesive potential. This study represents the first systematic quantification of hydroxyproline across sixteen fish species in the context of adhesive performance, extending previous reports limited to a few species. The findings provide a clear biochemical criterion for selecting fish skins as raw materials for bio-based adhesives [59]. Moreover, the established threshold offers a practical guideline for screening and optimizing collagen extraction for eco-friendly adhesive development. These insights underscore the potential of fish processing waste as a sustainable resource, advancing circular bioeconomy approaches while enabling the rational design of high-performance, collagen-based adhesives [61].

**Figure 4:**
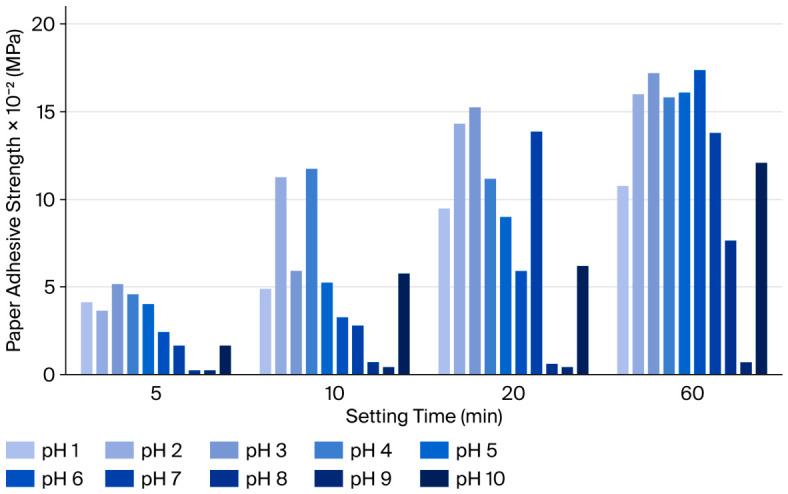
Effect of pH on the paper adhesion strength of milkfish skin adhesive. This figure was created in Microsoft® Excel® 2019 (Version 2205, Build 16.0.15225.20278, 64-bit) (Product ID: 00405-32438-96064-AAOEM).

**Effect of pH on paper adhesion of the milkfish skin adhesive.** As shown in Figure 4, paper bonding strength increased over time across all pH treatments. Bonding strength did not vary linearly with pH. At an early setting time of 5 min, the adhesive at pH 3 exhibited the highest bonding strength (5.12 × 10^−2^ MPa). After 60 min, adhesives at pH 6 (17.43 × 10^−2^ MPa) achieved comparable bonding strength to those at pH 3 (17.23 × 10^−2^ MPa). These results indicate that a lower pH accelerates initial adhesion, with pH 3 providing the fastest bonding. In contrast, a slightly higher pH (pH 6) achieves equivalent maximum adhesion over longer contact times. Therefore, optimizing paper bonding speed favors adjustment to pH 3, whereas maximizing long-term adhesion performance can be achieved at either pH 3 or pH 6, depending on application requirements. The observed variation in bonding strength with pH highlights the influence of protonation on collagen-based adhesive interactions [62]. At pH 3, the adhesive exhibited the fastest initial paper adhesion, likely due to enhanced hydrogen bonding and electrostatic interactions under acidic conditions, which promote rapid network formation. In contrast, adhesives at pH 6 achieved comparable ultimate bonding strength after prolonged setting (60 min), suggesting that slightly higher pH favors gradual network consolidation and stable adhesion. These findings indicate that pH modulates the kinetics of adhesion rather than the maximum achievable strength [63]. This behavior has practical implications for designing collagen–PVP biohybrid adhesives: adjusting to pH 3 optimizes initial tack and rapid bonding, whereas pH 6 can be employed when sustained adhesion over longer durations is required. Such tunability enhances the versatility of fish skin-derived adhesives for paper bonding applications. Moreover, the results underscore the interplay between physicochemical environment and functional performance in bio-based adhesives, providing a foundation for rational formulation strategies that balance adhesion speed and strength [64] (Figure 4).

**Adhesive performance of fish skin-derived adhesive sticks.** As shown in Table 3, the adhesive strength of glue sticks derived from fish skin at different setting times (5, 10, 20, and 60 min) showed no statistically significant differences when compared to cassava starch-based and commercial glue sticks, except at 60 min. However, adhesives formulated from natural biopolymers exhibited slightly lower bonding strength than commercial formulations. In particular, the milkfish skin adhesive stick demonstrated paper adhesion values approximately 1.15–1.35 times lower than the commercial control. The maximum adhesion strength of the fish skin glue stick was observed at a 60-min setting time, reaching 13.19 × 10^−2^ MPa. These findings suggest that while fish skin-derived glue sticks achieve comparable bonding performance to bio-based starch adhesives, there remains a modest difference in adhesion compared to synthetic commercial counterparts, particularly at extended setting times. This indicates that optimizing the formulation—such as by tuning polymer interactions or plasticizer content—could further enhance adhesion efficiency for sustainable adhesive applications. The adhesive performance of the milkfish skin collagen–PVP glue stick highlights an intrinsic trade-off between bio-based sustainability and mechanical strength. Although its paper adhesion was slightly lower than that of synthetic commercial glues, the performance remained within an acceptable range for practical use. The results align with prior studies [65,66] showing that biopolymer-based adhesives, while offering biodegradability and non-toxicity, often exhibit moderate adhesion due to lower polymer cross-link density and moisture sensitivity [65,66]. Nevertheless, the fish skin formulation demonstrated stable bonding over time, indicating adequate structural integrity and compatibility with cellulose substrates. Importantly, the use of fish processing by-products as a collagen source represents a significant advancement in sustainable adhesive development, reducing reliance on petrochemical polymers. Future optimization—such as enhancing polymer blending, adjusting pH, or incorporating natural cross-linkers—could further improve adhesion strength while preserving biodegradability, supporting the transition toward eco-friendly adhesive technologies.

**The physical properties of the milkfish skin-derived adhesive stick**, including color parameters, moisture content, melting point, pH, hardness, and peeling strength, as presented in Table 4, showed significant differences (*p* < 0.05) compared to the commercial glue stick, except for the a* value and pH. The adhesive exhibited a slightly alkaline nature, with a pH of approximately 10. Notably, the wet adhesive per unit area and dry adhesive per unit area of the milkfish skin adhesive were significantly higher than those of the commercial counterpart, while the initial adhesive performance was comparable, with a consistent setting time of 60 s. These results suggest that the collagen-based composition of the milkfish skin glue enhances adhesion strength under both wet and dry conditions, potentially due to the strong intermolecular hydrogen bonding and cohesive film formation inherent to collagenous matrices [67]. The superior wet and dry adhesive strength observed in the milkfish (*C. chanos*) skin-derived glue stick can be attributed to the intrinsic triple-helical collagen structure, which provides abundant hydroxyl, carboxyl, and amide functional groups capable of forming strong intermolecular hydrogen bonds with cellulose fibers in paper substrates [68]. The alkaline nature (pH ≈ 10) may further enhance peptide chain flexibility and interfacial wetting, improving penetration and cohesive film formation [67]. Compared to synthetic polymer-based commercial adhesives, the collagen matrix enables higher adhesion under humid or water-exposed conditions, indicating its robustness and biocompatibility [69]. However, its slightly softer texture and lower hardness compared to petrochemical-based formulations suggest a trade-off between biodegradability and mechanical rigidity. Overall, the findings demonstrate that fish skin-derived collagen adhesives can achieve comparable initial adhesion with superior long-term bonding performance, highlighting their potential as sustainable, bio-based alternatives to conventional glue sticks and aligning with global goals toward greener, safer adhesive technologies.

**Figure 5:**
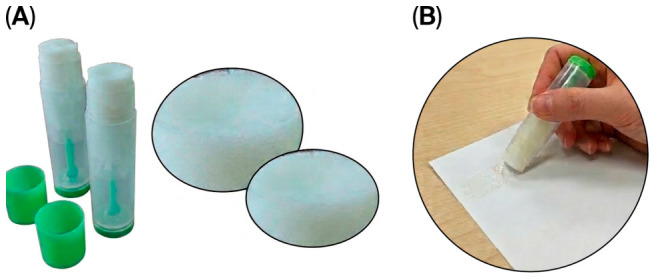
Prototype of collagen–PVP biohybrid adhesive in stick form. (**A**) Solid-state formulation demonstrating processability and handling characteristics. (**B**) Exposed adhesive surface showing the semi-solid structure of the polymer system.

**Consumer acceptance and functional performance of milkfish skin-derived adhesive stick.** As shown in Table 5, Consumer acceptance of the milkfish skin-derived adhesive stick was evaluated using QDA to assess its feasibility for practical application and market potential. The assessment included pre-use appearance (visual turbidity, surface smoothness, and structural homogeneity), in-use performance (application smoothness, application hardness, glue consumption per application, and coating uniformity), and post-use bonding performance (repositionability, bonding strength, and shape retention). When compared to a commercial glue stick, no significant differences (*p* > 0.05) were observed for most attributes, indicating comparable consumer-perceived quality. Exceptions were noted for application hardness, glue consumption per application, and shape retention, where the fish skin-derived adhesive exhibited slightly lower hardness, higher glue usage, and reduced shape retention. These findings suggest that the milkfish skin-based adhesive is largely acceptable to consumers, with minor trade-offs that can be optimized in formulation for commercial deployment (Figure 5).

The QDA demonstrated that the milkfish (*C. chanos*) skin-derived glue stick exhibited consumer-perceived attributes largely comparable to commercial adhesives across pre-use, in-use, and post-use parameters. Minor deviations in application hardness, glue consumption per application, and shape retention highlight trade-offs inherent to bio-based formulations, where enhanced biodegradability and sustainability can slightly compromise mechanical robustness [32]. These differences likely arise from the intrinsic viscoelastic properties of collagen matrices, which, while promoting adhesion through hydrogen bonding and cohesive film formation, yield a softer texture and greater material spread during application [67]. Despite these trade-offs, the adhesive maintains satisfactory performance for practical use, confirming its feasibility for commercial deployment [70]. Importantly, this work demonstrates that valorization of fish-processing waste into functional, consumer-acceptable adhesives is achievable, supporting circular economy principles and reducing reliance on petrochemical-based products. Such innovation underscores the potential of sustainable, bio-based adhesives to bridge environmental responsibility with performance-driven application in everyday materials [68,69].

**Shelf-life performance of the milkfish skin-based adhesive stick.** During storage at room temperature (0–104 days), the milkfish skin-based glue stick exhibited progressive changes in physical properties and sensory acceptance relative to commercial glue sticks. Moisture content decreased significantly from day 7 onward (64.72–42.58%, *p* < 0.05), accompanied by a gradual reduction in water activity (0.961–0.875), whereas commercial glue sticks showed no significant variation in either parameter (*p* > 0.05). These trends indicate moisture loss as a primary factor influencing storage stability in the bio-based formulation. Color analysis revealed a decrease in lightness (L*) over time, with increasing negative a* and positive b* values, suggesting mild chromatic changes during storage. In contrast, commercial glue sticks maintained stable color attributes. Sensory evaluation using a 15-point hedonic scale demonstrated a storage-dependent decline in acceptance scores for the milkfish skin glue stick, with stick hardness being the most affected attribute. Notably, overall liking remained statistically comparable to that of commercial glue sticks up to 49 days (*p* > 0.05). Considering both sensory score differences and physical stability, the milkfish skin-based glue stick exhibited a shelf life of at least 70 days, with most quality attributes remaining within acceptable limits. These findings highlight the inherent trade-off between sustainability and long-term stability in bio-based adhesives while demonstrating the practical feasibility of fish skin-derived glue sticks for short- to mid-term consumer use. The storage-related changes observed in the milkfish skin-based collagen–PVP glue stick are primarily governed by moisture–matrix interactions within the collagen network. Progressive moisture loss reduces bound water in the protein–polymer matrix, leading to decreased plasticization and enhanced intermolecular hydrogen bonding among collagen chains, which results in increased rigidity and reduced stick hardness during storage [71,72]. Bound water is essential for stabilizing the triple-helical structure of collagen; thus, dehydration can induce network contraction and alter viscoelastic behavior without fully disrupting adhesive bonding sites [73]. The hydrophilic nature of polyvinylpyrrolidone (PVP) enables hydrogen bonding with both collagen and water molecules, partially moderating moisture migration but not fully preventing dehydration over time [72,74]. Consequently, changes in mechanical and surface attributes are more pronounced than losses in adhesion performance. Despite this moisture sensitivity, adhesive functionality and consumer acceptance remain comparable to commercial glue sticks for short- to mid-term storage, highlighting an inherent sustainability–performance trade-off common to protein-based bioadhesives [31,74,75].

## 4. Conclusions

In this study, we demonstrate the feasibility of utilizing milkfish (*C. chanos*) skin, a seafood processing by-product, as a collagen source for the preparation of protein-based adhesive materials. Type I collagen was extracted and formulated with PVP to produce a biohybrid adhesive stick, and its physicochemical and adhesive properties were systematically characterized. The results indicate that moisture content and water activity strongly influence the physical stability of the collagen matrix during storage, likely through modulation of intermolecular hydrogen bonding and network mobility. The incorporation of PVP contributed to improved cohesion and peeling strength, suggesting favorable interactions between collagen chains and the synthetic polymer. Although gradual changes in hardness and moisture-related properties were observed over storage, the adhesive performance remained comparable to that of commercial glue sticks within an acceptable timeframe. Overall, the findings provide compositional and mechanistic insights into collagen–polymer systems derived from food by-products and support their potential for further development as functional biomaterials within the context of sustainable resource utilization. This study highlights collagen-rich by-products as tunable polymeric building blocks for biohybrid networks. The correlation between molecular interactions and macroscopic adhesion provides a framework for structure–property-driven material design. These insights may guide the development of protein–polymer systems for advanced and sustainable applications.

## Figures and Tables

**Table 1:** Characteristics of adhesive derived from milkfish (*C. chanos*) skin in comparison with animal hide and bone adhesives.

Characteristics	Adhesive Derived from Milkfish Skin Waste [Mean (Unit) ± SD; *n* = 3]	Animal Hide Adhesive ^1^	Bone Adhesive ^1^
Paper adhesive strength * (MPa × 10^−2^)	19.3 ± 0.3	–	–
Moisture (%)	97.26 ± 0.19	10–14	8.0–11
Ash (%)	0.061 ± 0.01	2.0–5.0	2.0–4.0
Fat (%)	0.342 ± 0.10	0.4–1.0	0.4–4.0
Protein (%)	1.214 ± 0.01	–	–
Carbohydrate (%)	1.127 ± 0.18	–	–
pH	3.35 ± 0.04	6.0–7.5	5.0–6.5
Viscosity (cP)	18,330 ± 4.62	3000–20,000	2500–9000
Residue after evaporation (%; *w*/*w*)	2.76	–	–
Yield (%)	68.82	–	–

^1^ modified from [43]; –: not reported; * Setting time at 60 min.

**Table 2:** Paper adhesive strength and hydroxyproline content of fish skin waste adhesives from various species.

Fish Species	Common Name	Paper Adhesive Strength * (Mean (MPa) ± SD × 10^−2^; *n* = 3)	Hydroxyproline Content (Mean (mg/g of Glue Weight) ± SD; *n* = 3)
*Chanos chanos*	Milkfish	19.3 ± 0.3 ^a^	554.1 ± 54.5 ^b^
*Oreochromis niloticus*	Nile tilapia	18.7 ± 0.3 ^a^	304.6 ± 49.6 ^de^
*Psettodes erumei*	Indian halibut	17.2 ± 0.2 ^b^	559.0 ± 85.8 ^b^
*Micronema bleekeri*	Whisker sheatfish	16.9 ± 0.2 ^bc^	445.7 ± 100 ^bc^
*Channa striata*	Striped snakehead	15.6 ± 0.2 ^cd^	231.1 ± 52.9 ^defg^
*Epiephelus malabaricus*	Areolated grouper	14.8 ± 0.4 ^d^	705.6 ± 38.1 ^b^
*Priacanthus tayenus*	Purple-spotted bigeye	11.9 ± 0.5 ^e^	213.7 ± 58.5 ^efg^
*Lethrinus lentjan*	Pink ear emperor	11.6 ± 0.5 ^ef^	251.9 ± 42.3 ^def^
*Oreochromis* sp.	Red tilapia	11.3 ± 0.0 ^ef^	357.9 ± 46.7 ^cd^
*Barbonymus gonionotus*	Java barb	10.5 ± 0.2 ^f^	698.4 ± 69.7 ^a^
*Lutjanus lineolatus*	Bigeye snapper	7.8 ± 0.2 ^g^	-
*Nemipterus hexodon*	Ornate threadfin bream	6.8 ± 0.2 ^g^	549.9 ± 148 ^b^
*Sphyraena jello*	Pickhandle barracuda	0	169.6 ± 21.9 ^fgh^
*Xenentodon cancila*	Freshwater garfish	0	42.87 ± 17.1 ^h^
*Liza vaigiensis*	Squaretail mullet	0	130.8 ± 37.0 ^fgh^
*Anabas testudineus*	Climbing perch	0	123.3 ± 6.60 ^fgh^
*Thunnus tonggol*	Longtail tuna	0	197.0 ± 15.9 ^efg^
*Rastrelliger brachysoma*	Short mackerel	0	115.2 ± 31.9 ^gh^
*Pomadasys hasta*	Lined silver grunt	0	119.1 ± 8.03 ^gh^
*Scomber japonicus*	Chub mackerel	0	178.6 ± 14.2 ^efg^

^a–h^ means in the same column, followed by different letters, are significantly different (*p* < 0.05); - did not analyze; * setting time of 60 min.

**Table 3:** Paper adhesive strength among adhesive sticks from milkfish skin waste, adhesive sticks from cassava starch, and commercial adhesive sticks.

Setting Time (min)	Paper Adhesive Strength [Mean (MPa × 10^−2^) ± SD; *n* = 3]		
	Adhesive Sticks from Milkfish Skin Waste	Adhesive Sticks from Cassava Starch	Commercial Adhesive Sticks
5	7.94 ± 0.46 ^Ba^	8.43 ± 0.47 ^Ba^	10.76 ± 0.66 ^Aa^
10	11.22 ± 0.46 ^Bb^	11.16 ± 0.32 ^Bb^	13.40 ± 0.48 ^Ab^
20	12.73 ± 0.63 ^Bb^	13.16 ± 0.64 ^Bc^	14.72 ± 0.56 ^Ac^
60	13.19 ± 0.74 ^Cc^	14.51 ± 0.77 ^Bd^	16.40 ± 0.44 ^Ad^

^A–C^ means in the same row, followed by different letters are significantly different (*p* < 0.05). ^a–d^ means in the same column, followed by different letters, are significantly different (*p* < 0.05).

**Table 4:** Physical characteristics of biohybrid collagen–polyvinylpyrrolidone (18%) adhesive sticks derived from milkfish (*C. chanos*) skin waste compared to commercial adhesive sticks for paper bonding.

Characteristics (Unit)	Content ((Mean) ± SD; *n* = 3)	
	Adhesive Sticks from Milkfish Skin	Commercial Adhesive Sticks
Color		
L*	79.8 ± 0.38 ^a^	81.6 ± 0.79 ^b^
a*	−1.72 ± 0.12 ^ns^	−1.83 ± 0.05 ^ns^
b*	2.23 ± 0.43 ^b^	1.61 ± 0.05 ^a^
moisture content (%)	65.6 ± 0.55 ^b^	60.3 ± 0.60 ^a^
melting point (°C)	81.9 ± 1.78 ^ns^	81.1 ± 1.29 ^ns^
pH	10.1 ± 0.02 ^ns^	10.2 ± 0.02 ^ns^
hardness (N)	0.56 ± 0.08 ^b^	1.10 ± 0.06 ^a^
peel strength (N)	1.72 ± 0.29 ^ns^	1.89 ± 0.31 ^ns^
initial adhesive (s)	60 ^ns^	60 ^ns^
wet adhesive per unit area (g/cm^2^)	2.79 × 10^−4^ ± 0.35 ^b^	2.29 × 10^−4^ ± 0.02 ^a^
dry adhesive per unit area (g/cm^2^)	1.16 × 10^−4^ ± 0.05 ^b^	0.65 × 10^−4^ ± 0.06 ^b^

L* represents lightness, ranging from 100 (white) to 0 (black). a* indicates redness (positive values) and greenness (negative values). b* indicates yellowness (positive values) and blueness (negative values). ^ab^ means in the same row, followed by different letters, are significantly different (*p* < 0.05). ^ns^ indicates no significant difference (*p* > 0.05).

**Table 5:** Sensory evaluation of biohybrid collagen–polyvinylpyrrolidone (18%) adhesive sticks derived from milkfish (*C. chanos*) skin waste compared to commercial adhesive sticks for paper bonding using quantitative descriptive analysis (QDA).

Sensory Attribute	Description	Scale (1–15)	Intensity ((Mean) ± SD; *n* = 10)	
			Adhesive Sticks from Milkfish Skin	Commercial Adhesive Sticks
Pre-use appearance				
Visual turbidity	Clarity or cloudiness of adhesive	1 = very clear, 15 = very turbid	7.12 ± 1.85 ^ns^	7.43 ± 2.48 ^ns^
Surface smoothness	Smoothness of stick surface	1 = very rough, 15 = very smooth	7.93 ± 1.13 ^ns^	8.24 ± 1.51 ^ns^
Structural homogeneity	Uniformity of internal structure	1 = very heterogeneous, 15 = very homogeneous	7.87 ± 1.82 ^ns^	8.47 ± 1.50 ^ns^
In-use performance				
Application smoothness	Ease of spreading glue on paper	1 = very difficult, 15 = very smooth	8.92 ± 1.45 ^ns^	7.99 ± 1.87 ^ns^
Application hardness	Resistance to deformation while applying	1 = very soft, 15 = very hard	6.91 ± 1.61 ^b^	8.48 ± 1.37 ^a^
Glue consumption per application	Amount of glue used to cover standard area	1 = very low, 15 = very high	7.87 ± 2.17 ^b^	6.35 ± 0.71 ^a^
Coating uniformity	Evenness of glue layer on paper	1 = very uneven, 15 = very uniform	8.36 ± 1.66 ^ns^	7.32 ± 2.34 ^ns^
Post-use bonding performance				
Repositionability	Ease of adjusting position after initial contact	1 = very difficult, 15 = very easy	6.47 ± 2.93 ^ns^	7.35 ± 2.06 ^ns^
Bonding strength	Adhesive performance after set time	1 = very weak, 15 = very strong	8.40 ± 2.62 ^ns^	7.35 ± 2.06 ^ns^
Shape retention	Ability to maintain shape after use	1 = very poor, 15 = very good	6.62 ± 1.20 ^b^	8.64 ± 1.45 ^a^

^a,b^ means in the same row, followed by different letters, are significantly different (*p* < 0.05). ^ns^ indicates no significant difference (*p* > 0.05).

## Data Availability

The original contributions presented in this study are included in the article. Further inquiries can be directed to the corresponding author.
